# Cell Reprogramming in Tumorigenesis and Its Therapeutic Implications for Breast Cancer

**DOI:** 10.3390/ijms20081827

**Published:** 2019-04-12

**Authors:** Pei-Yi Chu, Ming-Feng Hou, Ji-Ching Lai, Long-Fong Chen, Chang-Shen Lin

**Affiliations:** 1 School of Medicine, College of Medicine, Fu Jen Catholic University, New Taipei City 242, Taiwan; chu.peiyi@msa.hinet.net; 2 Department of Pathology, Show Chwan Memorial Hospital, Changhua 500, Taiwan; qoxoruby@gmail.com; 3 National Institute of Cancer Research, National Health Research Institutes, Tainan 704, Taiwan;; 4 Department of Surgery, Kaohsiung Medical University Hospital, Kaohsiung 807, Taiwan; mifeho@kmu.edu.tw; 5 Department of Medical Research, Show Chwan Memorial Hospital, Changhua 500, Taiwan; jichinglai@gmail.com; 6 Graduate Institute of Medicine, Kaohsiung Medical University, Kaohsiung 807, Taiwan; 7 Department of Medical Research, Kaohsiung Medical University Hospital, Kaohsiung 807, Taiwan; 8 Department of Biological Sciences, National Sun Yet-sen University, Kaohsiung 804, Taiwan

**Keywords:** breast cancer, cell plasticity, cell reprogramming, lineage conversion, mammary stem cells

## Abstract

Breast cancer is the most common malignancy in women worldwide and can be categorized into several subtypes according to histopathological parameters or genomic signatures. Such heterogeneity of breast cancer can arise from the reactivation of mammary stem cells in situ during tumorigenesis. Moreover, different breast cancer subtypes exhibit varieties of cancer incidence, therapeutic response, and patient prognosis, suggesting that a specific therapeutic protocol is required for each breast cancer subtype. Recent studies using molecular and cellular assays identified a link between specific genetic/epigenetic alterations and distinct cells of origin of breast cancer subtypes. These alterations include oncogenes, tumor suppressor genes, and cell-lineage determinants, which can induce cell reprogramming (dedifferentiation and transdifferentiation) among two lineage-committed mammary epithelial cells, namely basal and luminal cells. The interconversion of cell states through cell reprogramming into the intermediates of mammary stem cells can give rise to heterogeneous breast cancers that complicate effective therapies of breast cancer. A better understanding of mechanisms underlying cell reprogramming in breast cancer can help in not only elucidating tumorigenesis but also developing therapeutics for breast cancer. This review introduces recent findings on cancer gene-mediated cell reprogramming in breast cancer and discusses the therapeutic potential of targeting cell reprogramming.

## 1. Introduction

Embryonic stem cells (ESCs) are pluripotent stem cells that can differentiate into three germ layers: endoderm, mesoderm, and ectoderm. After the differentiation of ESCs into different germ layers, the resulting daughter stem/progenitor cells and terminally differentiated cells can be distinguished based on the differential expression of lineage- and tissue-specific markers. For example, *GATA4*, *FOXA2* (HNF-3β), *HNF-4A*, *SOX17*, and alpha fetoprotein are specifically expressed in the endoderm; *NES* (nestin), *SOX1*, *MAP2*, and *GFAP* are expressed in the ectoderm; and *TBXT* (Brachyury), *KDR* (FLK1), *VIM* (vimentin), and *FN1* (fibronectin) are expressed in the mesoderm. Many of these marker genes encode transcription factors (TFs) that are critical for cell fate specification. After lineage commitment, stem/progenitor cells usually undergo downward, lineage-specific differentiation and cannot go back to the stem-cell state. However, Takahashi and Yamanaka [1] introduced a cell reprogramming method that utilizes a combination of four TFs, namely *OCT4*, *SOX2*, *KLF4*, and *MYC* (OSKM), to convert differentiated fibroblasts back to an ESC-like state; the resulting cells are called induced pluripotent stem cells (iPSCs). This cell reprogramming method was proven to be successful in numerous cell types with various differentiation statuses and was applied in many research fields, including cancer research. For example, Corominas-Faja et al. [2] used OSKM to reprogram the MCF-7 human breast cancer cells into SOX2-overexpressing cancer stem cell (CSC)-like cells that exhibit activated mammalian target of rapamycin (mTOR) kinase activity. In addition, OSKM could reprogram MCF-10A cells, a non-tumorigenic human mammary epithelial cell line, into CSC-like cells, which express the stem-cell marker CD44 and feature enhanced malignancy [3].

In addition to the OSKM-mediated cell reprogramming of differentiated cells into iPSCs, many studies used single or a few lineage-specific factors, usually TFs, to directly convert one cell type into another. Such lineage switch is a process of direct reprogramming (DR) or transdifferentiation [4]. For example, Tani et al. [5] reported that a combination of three cardiac-specific TFs (*GATA4*, *MEF2C*, and *TBX5*) can directly reprogram fibroblasts into cardiomyocytes and that additional factors, such as microRNAs (miRNAs), cytokines, and epigenetic factors, can modulate this cardiac DR. In the cases of mammary and breast cancer cells, overexpression of *GATA3* or *NOTCH1* in mammary basal cells (BCs) can convert BCs to luminal cells (LCs) [6,7]. By contrast, forced expression of *TP63* reprograms LCs to BCs [8,9]. Such interconversion between mammary BCs and LCs demonstrates the cell plasticity of both epithelial lineages in the mammary gland. Because the normal development process and tumorigenesis of the mammary gland epithelium share similar signal pathways [10,11,12,13], study of mechanisms underlying lineage conversion or DR can not only illustrate the control of mammary gland development but also elucidate the tumorigenesis of breast cancer. Lineage interconversion may contribute to tumor heterogeneity and increase the number of breast cancer subtypes under oxidative and therapeutic stresses, which can complicate the curative therapy of advanced cancer [4,12,13]. Thus, a better understanding of cell reprogramming mechanisms in breast cancer can be helpful to unveil the potential therapeutic strategy to target different subtypes of breast cancer.

## 2. Epithelial Cell Lineages in the Mammary Gland and Subtypes of Breast Cancer

**Figure 1:**
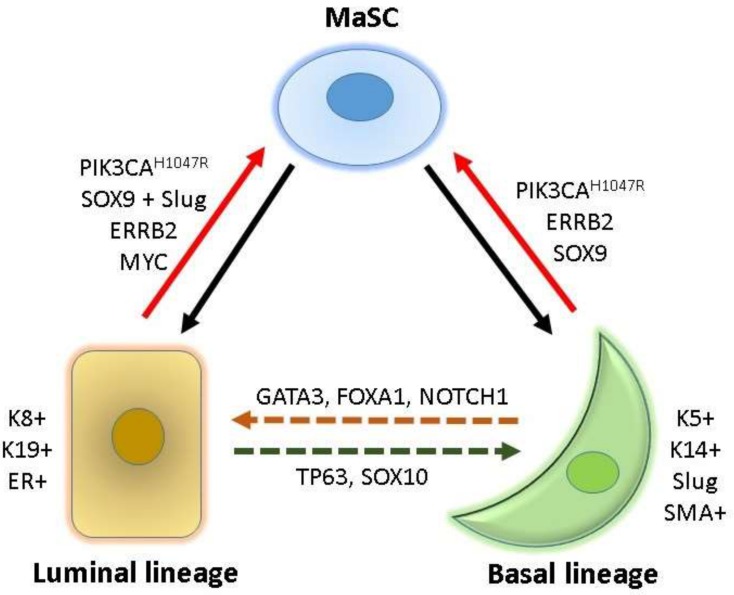
Cell hierarchy and cell reprogramming in the mammary tissue. Bipotent mammary stem cells (MaSC) can differentiate into luminal and basal lineages (indicated by solid back lines). Cytokeratin (K) 8, K19, and estrogen receptor (ER) are luminal lineage-specific markers, and K5, K14, Slug, and smooth muscle actin (SMA) are basal lineage-specific markers. Overexpression of lineage-specific factors may induce interconversion between luminal and basal lineages (dotted lines). Oncogene activation can reprogram lineage-committed basal and luminal cells into MaSC (red lines), which can give rise to heterogeneous breast cancers.

In mouse models, multipotent mammary stem cells (MaSCs) that express both basal (e.g., *Trp63*, cytokeratin 5 (*Krt5*), and *Krt14*) and luminal (e.g., *Notch1* and *Krt8*) signature genes can be found during embryonic development until day 14.5 (E14.5). After E17, only unipotent luminal and basal progenitors (LPs and BPs, respectively) are present in the mammary epithelium, which support tissue homeostasis throughout adulthood [7,8,14]. The mammary gland epithelium contains two major cell types, namely LCs (ductal and secretory alveolar cells) and BCs (myoepithelial cells); both these cell types are generated and maintained in adult mammary tissues by their own long-lived and unipotent LPs and BPs, respectively [15]. Another study found that, in adult mice, a small fraction of BPs can differentiate into both basal and luminal lineages, suggesting that a few BPs are bipotent MaSCs [16]. However, studies showed that, at least in postnatal mice, the homeostasis of the mammary gland epithelium is maintained by lineage-restricted unipotent LPs and BPs [17,18]. Nevertheless, both differentiated basal- and luminal-lineage mammary cells preserve the capacity to dedifferentiate into an intermediate bipotent MaSC state [19] and then develop into BCs or LCs [7,8] (Figure 1).

TFs are critical fate-determinants for the mammary development of basal and luminal lineages [20,21,22]. For the luminal differentiation from bipotent MaSCs, *NOTCH1*, *ELF5*, and *EHF* contribute to the development of LPs [7,9,22,23] and *GATA3*, *FOXA1*, and *ESR1* (estrogen receptor alpha) are critical for further differentiation into mature LCs [6,22,24]. *TP63*, *NF1*, and *SNAI2* (Slug) are required for the differentiation of the basal lineage [9,22]. In addition to TFs, other cell surface markers and lineage-specific molecules are useful for the identification and purification of various lineage-restricted cells from mammary tissues. For example, smooth muscle actin, KRT5, KRT14, and vimentin are specifically expressed in the basal lineage, and ESR1, progesterone receptor (PR), E-cadherin (CDH1), EPCAM, KRT8, KRT18, and KRT19 are predominant in the luminal lineage [20,21]. These lineage-specific makers and TFs are commonly used to classify and trace the cell of origin of various mammary epithelial and breast cancer cells (Figure 1).

Breast cancers are organized and constituted of heterogeneous mammary cell types in a hierarchy pattern. According to the histological expression of ERα, PR, and HER2/ERRB2, breast cancer patients are divided into three therapeutic groups: ER-positive, HER2, and triple-negative breast cancer (TNBC) who receive hormone therapy, anti-HER2 target therapy, and chemotherapy, respectively. According to their intrinsic transcriptional profiles, breast cancers can also be classified into five subtypes: luminal A, luminal B, HER2, TNBC/basal-like, and normal like [25,26,27,28]. Additional subtypes, such as claudin low, were identified through further analyses with the aid of more breast cancer cohorts [25,26,27,28]. These heterogeneous breast cancers exhibit different incidences, therapeutic responses, and prognoses [29]. However, the connection between breast cancer subtypes and the cell of origin during tumorigenesis is not fully elucidated. Several recent studies revealed that heterogeneous breast cancers can arise from different mammary cells through lineage interconversion and cell reprogramming; some examples and their therapeutic implications are given below.

## 3. Cancer Gene-Mediated Cell Reprogramming in Breast Cancer

Recent studies demonstrated that either the activation of an oncogene or the inactivation of a tumor suppressor gene plays a critical role in cell reprogramming of lineage-restricted BCs or LCs into bipotent MaSCs (Figure 1), which may serve as tumor-initiating cells (TICs) or CSCs and confer heterogeneous breast cancers with diverse cells of origin (Table 1) [12,13]. Understanding the actions of these cell reprogramming factors in different mammary epithelial cells can facilitate the elucidation of mechanisms underlying the tumorigenesis of various breast cancer subtypes and the development of specific therapeutics for heterogeneous breast cancers.

### 3.1. PIK3CA

*PIK3CA* is one of the most frequently mutated genes in human breast cancer with distinct subtypes [30], implying the contribution of the *PIK3CA* mutation to breast cancer heterogeneity. The most recurrent activating mutation of *PIK3CA* in breast cancers is H1047R, which causes constitutive activation of the phosphatidylinositol 3-kinase (PI3K) pathway and proliferation of heterogeneous mammary epithelial cells [31,32,33]. In 2015, two independent studies used lineage tracing mouse models to show that the oncogenic expression of *Pik3ca*^H1047R^ in lineage-committed BCs or LCs can reprogram these differentiated cells into MaSCs and give rise to heterogeneous types of breast cancers [34,35]. The expression of *Pik3ca*^H1047R^ can also interconvert BCs into LCs and LCs into BCs. Transcriptional profiling revealed that *Pik3ca*^H1047R^ clearly rewires gene expression signatures in these cells, by which the switches of cell-identity signatures are correlated with the cells of origin and tumor types developed. These results demonstrate that the *PIK3CA*^H1047R^ mutation can initiate heterogeneous breast cancers by triggering the MaSC genetic program in differentiated BCs and LCs, thus providing new insights into the development of therapeutic strategies for PI3K-related breast cancers.

### 3.2. MYC

*MYC* is a member of OSKM acting on cell reprogramming [1] and is also a frequently deregulated oncogene in breast cancers, particularly in the basal-like subtype [30,36,37]. Poli et al. [38] reported that *MYC* acts as an oncogenic reprogramming factor to convert *TERT*-immortalized human mammary epithelial cells and luminal breast cancer cells into the basal/stem cell-like state and gives rise to TICs favoring the onset of mammary tumorigenesis. Overexpression of *MYC* induces alternative transcriptional and epigenetic programs, through which luminal lineage-specific enhancers and TFs, such as *GATA3* and *ESR1*, are suppressed. By contrast, *MYC* promotes the activation of de novo enhancers, which support the activation of oncogenic pathways (e.g., *WNT* and *EGFR*) and the onset of a stem cell-like state, represented by enhanced mammosphere formation [38]. Therefore, the luminal differentiation program is suppressed and mammary tumors emerge. These data suggest that *MYC* induces mammary tumorigenesis through cell reprogramming, which can be attributed to *MYC*-mediated activation of oncogenic enhancers and, meanwhile, repression of luminal fate-specific enhancers. Moreover, a mouse model with overexpression of *Myc* together with *Pik3ca*^H1047R^ endows long-term tumorigenicity and enhanced metastatic potential. Furthermore, metadata analyses indicate that oncogenic pathways activated by *MYC*-regulated enhancers are associated with a poor prognosis of patients with basal-like breast cancers [38]. Therefore, targeting *MYC* and *MYC*-regulated enhancers or oncogenic pathways represents potential therapeutic strategies for basal-like breast cancers.

### 3.3. ERBB2 and Polyomavirus Middle T

Basal-like breast cancers can arise from LCs with the oncogenic activation of *ERBB2* signaling or the expression of the polyomavirus middle T (PyMT) antigen [39]. Hein and colleagues [39] used the LC-restricted avian retroviral vector and in vivo tracing experiments to show that the lineage-specific expression of *ERBB2* or PyMT in committed LCs can induce these cells to generate mammary tumors derived from additional cellular lineages, revealing that either *ERBB2* or PyMT induces the plasticity of LCs during tumorigenesis. These results support the notion that the origin of breast cancer heterogeneity can arise from the activation of oncogenic signaling in the committed luminal lineage in addition to from multipotent stem cells.

### 3.4. TP53 and BRCA1

Similar to gain of function in oncogenes, the loss of function in tumor-suppressor genes may modulate the consequence of cell reprogramming in breast cancer. *TP53* is a roadblock for OSKM-induced cell reprogramming, and inactivation of *TP53* significantly increases the reprogramming efficiency of differentiated cells into iPSCs [40,41,42,43]. Inactivation of the *TP53* pathway is frequently observed in breast cancer, especially the basal-like TNBC [30], and is correlated with gene expression signatures of ESCs and MaSCs [44]. Zhang et al. [45] used a murine model to identify a Lin^−^CD29^H^CD24^H^ subset of TICs that can generate heterogeneous breast cancers in the absence of *Trp53*. The Lin^−^CD29^H^CD24^H^ subpopulation may have arisen from a bipotent MaSC [45]. A recent study reported that *MYC* is a target of *TP53*, whose inactivation leads to *MYC* activation and increases mammary cell plasticity [46]. These studies demonstrate the important role of *TP53* in guarding the identity of mammary cells and preventing the reprogramming of differentiated mammary cells into MaSCs and subsequent tumorigenesis.

*BRCA1* is a tumor-suppressor gene involved in transcriptional regulation and DNA repair [47]. Breast cancer patients with hereditary *BRCA1* mutations usually develop basal-like tumors. Deleting the *Brca1* gene in mouse luminal lineage cells generates tumors that are similar to human *BRCA1* breast cancer and sporadic basal-like breast cancers according to histological and transcriptional profiling analyses. By contrast, tumors derived from *Brca1*-defective BCs are histologically unlike human *BRCA1* or sporadic basal-like breast cancers [48]. These results indicate that basal-like breast cancer can arise from the luminal lineage instead of the basal lineage. Another study showed that *BRCA1*- and *FANCD2*-mediated DNA inter-strand crosslink repair controls the epithelial-to-mesenchymal transition (EMT) and the dedifferentiation of mammary epithelial cells [49]. These results suggest that *BRCA1*-mediated functions play a crucial role in modulating luminal–basal fate transition, EMT, and cell reprogramming.

### 3.5. SOX10

Based on the findings of the assay for transposase-accessible chromatin with high-throughput sequencing (ATAC-seq) and transcriptional profiling, the TF Sox10 was determined to be a critical fate determinant for cell state interconversion during mammary development [50]. SOX10 is expressed in mouse and human breast cancers where tumor cells highly expressing SOX10 exhibit characteristics of MaSCs, EMT, and neural crest cells. Overexpression of SOX10 can reprogram fibroblasts into a neural crest cell-like state [51], supporting the role of SOX10 in regulating cell state plasticity. The results of RNA-sequencing (RNA-seq) and chromatin immunoprecipitation-sequencing (ChIP-seq) performed in breast cancer cells revealed that SOX10 contributes to the expression of genes related to EMT, stem/progenitor cells, and neural crest cells, and suppresses genes related to epithelial cell differentiation and apoptosis. A single-allele deletion in the *Sox10* gene could significantly delay tumor development in a mouse model. In addition, overexpression of Sox10 induces the EMT of mammary epithelial cells [52]. These data demonstrate the critical role of SOX10 in regulating cell state plasticity and mammary tumorigenesis.

Taken together, these findings demonstrate that the activation of oncogenes and the inactivation of tumor-suppressor genes in lineage-restricted mammary epithelial cells can reprogram these cells into different lineages and the MaSC-like state, supporting the development of breast cancers and the evolution of tumor heterogeneity [12].

## 4. Targeting Cell Reprogramming for Breast Cancer Therapy

Through identifying critical cell reprogramming factors that contribute to tumorigenesis and tumor heterogeneity in breast cancer, researchers can develop therapeutic strategies to specifically target these cell reprogramming factors, which may improve breast cancer treatment (Table 2).

Recently, Wang et al. described an engineered MYC-fusion protein drug containing a dominant-negative MYC peptide (OmoMYC) and a functional penetrating “Phylomer” peptide (FPPa), with an improved drug delivery efficiency [53]. FPPa-OmoMYC triggers apoptosis in TNBC but not in non-tumorigenic cells. Gene expression signatures induced by FPPa-OmoMYC are different from those of *MYC*-induced cell reprogramming, which include transcriptional, metabolic, and apoptotic processes. FPPa-OmoMYC also synergistically enhances the efficacy of chemotherapeutic agents, including docetaxel, doxorubicin, and cetuximab, in the killing of breast cancer cells. Implementation of FPPa-OmoMYC and its derivatives in future clinical trials is expected for the treatment of *MYC*-associated breast cancer, such as basal-like TNBC.

Because PI3K signaling is activated at a high frequency in breast cancer [30] and contributes to cell reprogramming as described above [34,35], many therapeutics were developed to inhibit PI3K signaling [54]. For example, ER-positive breast cancers induced by estrogen in *Pik3ca*^H1047R^ mice are sensitive to the PI3K inhibitor BYL719 (alpelisib) in combination with BH3 mimetics, which inhibit *BCL2* family members [55]. However, either BYL719 or BH3 mimetic alone does not efficiently suppress estrogen-induced *Pik3ca*^H1047R^ breast cancer. These results indicate that the anti-apoptosis function of *BCL2* family members is important for cell survival during *PIK3CA*^H1047R^-induced cell reprogramming toward ER-positive breast cancer, consistent with apoptosis being a barrier for cell reprogramming [56,57]. Therefore, the combined inhibition of anti-apoptosis and cell reprogramming factors may improve the efficacy of anticancer therapy.

In addition to directly targeting cell reprogramming factors, manipulation of other fate-determinant TFs or lineage-committed molecules may also inhibit oncogenic cell reprogramming and tumorigenicity, because forced expression of such lineage-specific factors can induce differentiation of oncogenic MaSCs or CSCs, thus reducing tumorigenicity. For example, *KRT19* is dominantly expressed in the mammary epithelium of the luminal lineage. Overexpression of *KRT19* in KU-CSLC cells, a breast cancer stem-like cell line, significantly reduces CSC properties, whereas knockdown of *KRT19* in MDA-MB-231 TNBC cells increases tumor growth, sphere formation, cell migration, and drug resistance to doxorubicin [58]. These results suggest that overexpression of luminal-specific *KRT19* can reprogram basal-like TNBC cells and mammary CSCs into less aggressive and drug-sensitive states.

GATA3 is a master TF that induces mammary luminal differentiation [6,24] and is usually not expressed in basal-like breast cancers. Several studies showed that overexpression of *GATA3* reduces the tumorigenicity, EMT, and metastasis of basal TNBC cell lines, such as MDA-MB-231 and Hs578T [59,60,61,62]. In a mouse model of PyMT-induced breast cancer, overexpression of *Gata3* in late-stage tumors induces tumor differentiation and suppresses tumor dissemination [63]. GATA3 inhibits the expression of the metastasis-promoting genes *ID1/-3*, *KRTHB1*, *LY6E*, and *RARRES3* but upregulates the inhibitors of metastasis including the deleted in liver cancer 1 (*DLC1*) and progestagen-associated endometrial protein (*PAEP*) [59]. In addition, the expression of mesenchymal genes (vimentin, N-cadherin, and *MMP9*) is repressed, and the epithelial gene E-cadherin is activated by GATA3 [60]. GATA3 also inhibits the expression of lysyl oxidase, a metastasis-promoting and matrix-remodeling protein, through promoter methylation, and reprograms basal TNBC cells into a less aggressive phenotype [61]. Therefore, GATA3 increment in tumor cells may be a helpful method to treat basal-like TNBC.

FOXA1 plays a crucial role in the regulation of luminal differentiation and is not expressed in the basal subtype of breast cancer. Kong and colleagues [64] showed that overexpression of *FOXA1*, together with *GATA3* and *ESR1* in the TNBC cell lines MDA-MB-231 and BT-549, induces cell reprogramming of these cells into estrogen-responsive luminal-like cells, which are susceptible to hormone therapy. By contrast, inhibition of the expression of *FOXA1* in luminal breast cancer cell lines, such as MCF7 and T47D, and SKBR3 not only leads to silencing of luminal genes but also causes the induction of basal genes and enhanced cell migration and invasion, which represent the basal phenotype [65]. Therefore, although the targeting of *FOXA1* is proposed as a strategy to treat luminal types of breast cancers, this approach can result in cells reprogramming into more aggressive cancers [66,67]. There is still a need to determine strategies to precisely control the pros and cons of the utility of cell reprogramming and cell fate-determinant factors in cancer therapy.

In addition to PI3K inhibitors, Yuan et al. [68] screened a kinase inhibitor library and demonstrated that treatment with Rho-associated protein kinase (ROCK) and mTOR kinase inhibitors could reprogram TNBC cells into *NANOG*-expressing stem/progenitor cells. This result is supported by the finding of previous studies that ROCK inhibition promotes ESC survival [69] and mTOR inhibition prevents epithelial stem cell senescence [70]. ROCK and mTOR inhibitor-induced stem/progenitor cells can be differentiated into terminal adipogenic cells. These fat-like cells exhibit a gene expression profile similar to that of normal adipocytes and show reduced tumorigenicity in in vitro and in vivo assays. These results support the notion that induction of cancer cells undergoing differentiation can be a therapeutic strategy to treat breast cancer [71]. However, there are still obstacles for applying this reprogramming and differentiation approach in cancer patients, because we cannot ensure whether reprogramming-derived stem/progenitor cells will show a normal or less aggressive phenotype; in addition, it is difficult to know the right time window to induce the differentiation of these stem/progenitor cells in vivo [4]. Nevertheless, the induction of cancer cell reprogramming and differentiation is worthy of development in the future.

## 5. Epigenetic Perspectives on Breast Cancer Cell Reprogramming and Therapy

Although all cells derived from a fertilized egg have the same genetic information, descendent cells, including ESCs, and all differentiated cells in different lineages have distinct chromatin configurations and epigenetic states, which can be represented by specific posttranslational modifications (PTMs) of histones or histone codes. These differences define a unique phenotypic characteristic for each cell type and become a barrier to prevent switching between different cell lineages and states. Similarly, cancer cells and their normal counterparts share similar genetics (except for tumorigenic alterations) but exhibit considerably different epigenetic features. Thus, cell reprogramming and tumorigenesis, both of which change cell states and chromatin configurations, require overcoming epigenetic barriers present among the hierarchies of stem/progenitor cells and various cell lineages, as well as between normal and cancer cells [72]. It is important to better understand mechanisms underlying epigenetic reprogramming and chromatin remodeling between MaSCs/progenitors and basal/luminal lineages, which can be helpful in developing favorable therapeutic strategies for treating aggressive breast cancers [73].

During the cell reprogramming of differentiated cells into stem cells, lineage-restricted genes are gradually silenced and chromatin regions containing these genes are packaged into a more compact, closed configuration and cannot be accessed by TFs, RNA polymerase, and other cellular factors. By contrast, reprogramming factors, such as OCT4, SOX2, and KLF4, which are pioneer TFs, can access their target DNA sequences and enhancers and release the highly-packaged chromatin into an open state, allowing gene expression [73]. Chromatin regions spanning active enhancers usually feature monomethylation of histone H3 at lysine 4 (H3K4me1) and acetylation of histone H3 at lysine 27 (H3K27ac), which can be examined using ChIP assays. Therefore, changes in chromatin states or chromatin remodeling during cell reprogramming are correlated with changes in histone PTMs and gene expression profiles, through which one can distinguish between different cell identities accordingly. These changes can be genome-wide and comprehensively examined using recent advanced DNA sequencing technologies, such as RNA-seq, ChIP-seq, and ATAC-seq [73]. Integral data from RNA-seq, ChIP-seq, and ATAC-seq can generate high-resolution results to show genetic and epigenetic changes throughout the genome, allowing us to decipher more precisely the controlling mechanism for the cell reprogramming of breast cancers.

For example, using the aforementioned three techniques, Poli and colleagues [38] demonstrated that the *MYC*-induced cell reprogramming of mammary cells is linked to de novo activation of enhancers, which are enriched for the pioneer TFs of FOX- and SOX-family members and can derive stem/progenitor cell transcriptional programs and stem/progenitor cell phenotypes. Meanwhile, MYC induces a downregulation of lineage-specific TFs, such as *GATA3* and *ESR1*, which leads to decommissioning of luminal-specific enhancers. Similarly, Tu et al. [74] used ChIP-seq and RNA-seq approaches to further show that MYC represses the expression of tumor-suppressor genes, such as *CDKN1A*, *GADD45A*, and *HMOX1*, through interacting with G9A (EHMT2), a histone methyltransferase that causes H3K9 methylation and gene repression in breast cancer cells. Importantly, inhibition of G9A unleashes MYC-mediated suppression of these tumor-suppressor genes and reduces tumorigenicity, particularly in basal-like breast cancers. These results not only unveil molecular mechanisms underlying selective gene activation or repression by MYC but also identify a drug target for MYC-driven basal breast cancers. In addition to the MYC–G9A interaction, many cross-talks between cell reprogramming factors and chromatin remodelers were identified in breast cancer [50,73,75,76,77]; however, there might be many additional such networks that require further investigation.

## 6. Conclusions

Taking the advantages of these comprehensive and high-resolution genome-wide analyses including single-cell sequencing [78,79], we can uncover an increasing number of relationships between the cell reprogramming of lineage-committed mammary epithelial cells and the tumorigenesis of heterogeneous breast cancers [22,50,73,80,81]. In addition, by performing these analyses, many candidate therapeutic targets can be found and examined for improving the treatment of breast cancer patients. In conclusion, recent works using new technologies to decipher cell reprogramming mechanisms in breast cancer can extend our knowledge on the formation of breast cancer heterogeneity and speed up the development of therapeutic strategies.

## Figures and Tables

**Table 1:** Cell reprogramming factors in the tumorigenesis of breast cancer.

Reprogramming Factor	Action^2^	Target Cells	Consequence	Reference
*PIK3CA*	+	BC^3^, LC^4^	LC, BC, MaSC^6^, tumor	[34,35]
*MYC*	+	IMEC^5^, LC	BC, MaSC, tumor	[38]
*ERBB2*	+	LC	BC, MaSC, tumor	[39]
PyMT^1^	+	LC	BC, MaSC, tumor	[39]
*SOX10*	+	LC	BC, MaSC, NCC^7^	[50]
*SOX9* + Slug	+	BC, LC	MaSC, EMT^8^	[19]
*TP53*	-	LC	BC, MaSC, tumor	[44–46]
*BRCA1*	-	LC	BC, tumor	[48]
*FOXA1*	-	LC	BC, tumor	[65]

^1^ Polyoma virus middle T antigen; ^2^ activation (+) or inactivation (-) during cell reprogramming; ^3^ basal-lineage cells; ^4^ luminal-lineage cells; ^5^ *TERT*-immortalized mammary epithelial cells; ^6^ mammary stem/progenitor cells; ^7^ neural crest cells; ^8^ epithelial to mesenchymal transition.

**Table 2:** Targeting cell reprogramming for breast cancer treatment.

Target	Tumor Type	Action of Inhibition	Reference
PI3K, *BCL2* family	ER-positive^1^	Small molecule	[54,55]
*MYC*	TNBC^2^	Peptide	[53]
*KRT19*	CSC^3^, TNBC, Doxo^4^-resistant	Cell-fate determinant	[58]
*GATA3*	TNBC	Cell-fate determinant	[59–63]
*FOXA1*, *ESR1*, *GATA3*	TNBC	Cell-fate determinant	[64]
*ROCK*, *MTOR*	TNBC	Small molecule^5^	[68]
*G9A/EHMT2*	TNBC	Small molecule	[74]

^1^*PIK3CA*^H1047R^-driven tumor; ^2^ triple-negative breast cancer; ^3^ cancer stem cells; ^4^ doxorubicin; ^5^ subsequent differentiation into adipocytes.
